# The interaction of early life factors and depression-associated loci affecting the age at onset of the depression

**DOI:** 10.1038/s41398-022-02042-5

**Published:** 2022-07-25

**Authors:** Yujing Chen, Chuyu Pan, Shiqiang Cheng, Chun’e Li, Huijie Zhang, Zhen Zhang, Jingxi Zhang, Yao Yao, Peilin Meng, Xuena Yang, Li Liu, Bolun Cheng, Yumeng Jia, Yan Wen, Feng Zhang

**Affiliations:** 1 Key Laboratory of Trace Elements and Endemic Diseases of National Health and Family Planning Commission, School of Public Health, Health Science Center, Xi’an Jiaotong University, Xi’an, China; 2 Department of Psychiatry, The First Affiliated Hospital of Xi’an Jiaotong University, Xi’an, China

**Keywords:** Genomics, Depression, Scientific community

## Abstract

Multiple previous studies explored the associations between early life factors and the age at onset of the depression. However, they only focused on the influence of environmental or genetic factors, without considering the interactions between them. Based on previous genome-wide association study (GWAS) data, we first calculated polygenic risk score (PRS) for depression. Regression analyses were conducted to assess the interacting effects of depression PRS and 5 early life factors, including felt hated by family member (*N* = 40,112), physically abused by family (*N* = 40,464), felt loved (*N* = 35633), and sexually molested (*N* = 41,595) in childhood and maternal smoking during pregnancy (*N* = 38,309), on the age at onset of the depression. Genome-wide environment interaction studies (GWEIS) were then performed to identify the genes interacting with early life factors for the age at onset of the depression. In regression analyses, we observed significant interacting effects of felt loved as a child and depression PRS on the age at onset of depression in total sample (*β* = 0.708, *P* = 5.03 × 10^−3^) and males (*β* = 1.421, *P* = 7.64 × 10^−4^). GWEIS identified a novel candidate loci interacting with felt loved as a child at *GSAP* (rs2068031, *P* = 4.24 × 10^–8^) and detected several genes with suggestive significance association, such as *CMYA5* (rs7343, *P* = 2.03 × 10^–6^) and *KIRREL3* (rs535603, *P* = 4.84 × 10^–6^) in males. Our results indicate emotional care in childhood may affect the age at onset of depression, especially in males, and *GSAP* plays an important role in their interaction.

## Introduction

Depression is a widespread chronic medical illness often presenting with physical symptoms [1]. Over the past several decades, the amount of people with depression have increased leading to a growing concern in mental health research around the world [2]. According to the report of WHO, the global population suffering from depression was estimated to be 322 million, accounting for 4.4% of the global population that resulting in a surge in suicide rates as well as a huge social and economic burden [3].

Depression is a highly heterogeneous syndrome [4]. Various factors including different age at onset of the depressive episode, varying symptom profiles, different levels of symptom severity, and duration may contribute to clinical heterogeneity [5]. The age at onset of depression is the main reason for the clinical heterogeneity of depression [6]. Children having parents with early onset major depressive disorder (MDD) (<30) are 13 times more likely to develop early onset MDD than children in the control group [7]. Furthermore, early onset (<30) MDD has a higher heritability (*h*^*2*^ = 0.70), and late onset (>30) MDD is estimated to be much lower (*h*^*2*^ = 0.10) [8]. Early-onset depression and late-onset depression may have different pathogenesis [9]. It has long been suggested that early onset MDD is associated with increased risk of the disorder in first degree relatives, while late onset MDD was associated with increased familial risk of vascular disease [6, 10]. With increasing age, the course of depression became worsens. In a recent large cohort study of adults aged 18–88 years, people aged 70 and above experienced greater symptom severity compared with younger adults [11]. Therefore, the age at onset of the depression is important for the development of depression.

The early life factors of the newborn are crucial in the adaptation to (potentially life-long) environmental exposures. Relative to adulthood, neurodevelopment during childhood and adolescence is more plastic and susceptible to programming influences from stressful environmental and social contexts [12]. For example, childhood trauma exposure is associated with altered brain structure and function [13]. Altered hypothalamic-pituitary-adrenal (HPA) axis regulation and secondary regional brain structure changes in children exposed to emotional abuse, sexual abuse, and aggressive families [14]. Early life stress affects hippocampal neurogenesis, increases depressive-like behavior, and causes mild metabolic imbalance in early adulthood (2 months) [15].

Further, early life factors may modify the role of genetic polymorphism by providing the necessary substrates for the development of human disease or influencing the effect of a specific gene [16]. For example, individual genotypic variations interacting with early life stress may contribute to variability in clinical outcomes [17]. Alterations in humans with early life stress experiences include glucocorticoid resistance. Feedback inhibition of the HPA axis response via the glucocorticoid receptor (GR) signaling is significantly lower in patients with MDD, and cytosolic *FKBP5* gene expression is induced via GR activation [18]. Laboratory animals exposed to cigarette smoke in utero experience structural changes to their serotonin system that are associated with reduced serotonin levels and last through adulthood [19]. Low serotonin level is an important precursor to behaviors that are consistent with depression in animals and depression in humans [20].

The age at onset of depression is under genetic control, and genetic variants associated with MDD may differ according to age at onset of depression. Genome-wide association study (GWAS) only calculates the association between a single SNP and phenotype, which could easily lead to a decline in the interpretation of phenotypes affected by multiple genetic variants in complex diseases. However, previous studies on age at onset of depression merely focused on the effects of environmental or genetic factors on the risks, usually without considering the interaction between them.

Utilizing the UK Biobank cohort, we first calculated polygenic risk score (PRS) for depression based on previous depression GWAS summary data. The interacting effects of depression PRS and early life factors (e.g., childhood trauma exposure, mother smoking during pregnancy) on age at onset of the depression were tested through regression analysis. Then, genome-wide environment interaction studies (GWEIS) were performed to identify potential genetic loci × early life factors interacting effects on the risk of age at onset of the depression based on the regression analyses results.

## Methods

### UK Biobank cohort

The analysis data of study individuals were extracted from the UK Biobank health resource (https://www.ukbiobank.ac.uk/). UK Biobank is a very large prospective cohort study involving approximately 500,000 people aged 40–69, aiming to allow detailed investigations of the genetic and non-genetic determinants of the diseases of middle and old age [21]. It includes a range of phenotypic data such as measures of physical activity, blood, saliva, and urine biomarkers.

The genetic data contains genotypes of 488,377 participants which were assayed using the UK BiLEVE Axiom array and UK Biobank Axiom array. Marker-based quality control was performed by using statistical tests designed primarily to check for consistency of genotype calling across experimental factors to identify poor quality markers. SNPs with calling rate <98.5%, MAF < 0.01 were removed. Samples with calling rate <98.0% and mismatch between inferred sex and self-reported sex were removed. Imputation was carried out by IMPUTE4 (https://jmarchini.org/software/). Details of the array design, genotyping, and quality control procedures have been described previously (29). All data usage in this article was approved by UK Biobank (application 46478) and the Ethics Advisory Committee (EAC).

### Phenotypes definition of instrumental variable

Traumatic events during childhood, including felt hated by family member as a child (Data-Field 20487), physically abused by family as a child (Data-Field 20488), felt loved as a child (Data-Field 20489), and sexually molested as a child (Data-Field 20490), were collected from the responses of UK Biobank online “Thoughts and Feelings” mental health questionnaire: “When I was growing up …”, (a) I felt that someone in my family hated me, (b) people in my family hit me so hard, (c) I felt loved and, (d) Someone molested me (sexually) by choosing “Never true”, “Rarely true”, “Sometimes true”, “Often”, “Very often true”, and “Prefer not to answer”. The individuals with answers “Often” and “Very often true” were selected as cases, and with answers “Never true” and “Rarely true” were selected as controls. The subjects with answers “Sometimes true” and “Prefer not to answer” were excluded from this study. Maternal smoking during pregnancy (Data-Field 1787) was collected by asking “Did your mother smoke regularly around the time when you were born?” by choosing “Yes”, “No”, “Do not know” and “Prefer not to answer”. The subjects whose answers are “Do not know” and “Prefer not to answer” were excluded from this study.

### Phenotypes definition of outcome variable

Age at onset of depression (Data-Field 20433) for participants was collected by asking “how old you the first time feel you had a period of two weeks like prolonged feelings of sadness, depression or loss of interest in normal activities?”.

### GWAS dataset of depression

Depression-associated SNP set was derived from a previous GWAS [22]. Briefly, they performed stringent filtering on imputed variants (version 2) used for GWAS removing variants not among the 33,619,058 variant sites in the Haplotype Reference Consortium75 (HRC) panel, all insertions and deletions, and multi-allelic SNPs. SNPs with imputation INFO score above 0.9 and minor allele frequencies (MAF) > 5% were used to estimate genetic correlations. Sample filtering removed samples that were not included in the UK Biobank full release principal component (PC) analysis, then selected samples who self-reported ‘White British’ and have very similar genetic ancestry based on a principal components analysis of the genotypes.

### PRS Analysis of depression

Using the depression-associated SNPs from the GWAS datasets (*P* < 5 × 10^−8^), the PRS of depression for each individual was calculated as the sum of the risk allele they carried, weighted by the effect size of the risk allele [22]. The PRS of depression was computed by PLINK2.0, according to the formula:

$${\rm{PRS}}_n = \mathop {\sum}\nolimits_{i = 1}^l {E_iD_{\rm{in}}}$$

Let PRS_*n*_ denote the PRS value of depression for the nth subject; *l* and *i* denote the total number of sample size and genetic markers, respectively; *Ei* is the effect parameter of risk allele of the significant SNP associated with depression, which was obtained from the GWAS of depression and *D*_in_ denotes the dosage of the risk allele of the *i*th SNP for the *nth* individual (0 is coded for homozygous protective genotype, 1 for heterozygous and 2 for homozygous polymorphic genotypes) [23].

### Statistical analysis

The associations between early-life factors and age at onset of the depression were estimated through regression analysis. Specifically, linear regression analysis was conducted to test the associations between age at onset of depression and early life factors, depression PRS as well as their interactions in total individuals, female and male, respectively. All analyses were performed using R3.5.3 software, according to the formula:

$${{{{Y}}}} = \beta _0 + \beta _1G + \beta _2E + \beta _3G \ast E$$

Let *G* denote the depression PRS; *E* denote each early-life factor, respectively. In this regression analysis, age, sex, the first 10 PCs of population structure, Townsend deprivation index (TDI), smoking frequency, and alcohol use were adjusted as covariates. *P*-values 0.05 indicated the association was significant.

### Genome-wide environment interaction studies (GWEIS)

Using the generalized linear regression model of PLINK 2.0, GWGEIS were conducted to explore the interaction effects between SNPs and early life factors on the risk of age at onset of the depression in UK Biobank cohort. SNPs with call rate < 90%, MAF < 0.01 and Hardy-Weinberg equilibrium deviations *P* < 0.001 were removed. Age, sex, smoking frequency, alcohol use, TDI, and 10 principal components of population structure were used as covariates. A significance threshold was set as *P* = 5.0 × 10^−8^ for genome-wide by environment interaction effects. The suggestive significance threshold was set as *P* = 5.0 × 10^−6^.

## Results

### Descriptive characteristics of study participants

With age at onset of depression status as the outcome variable, a total of 35,633–41,595 participants answered the childhood traumatic events related questions, 61.92%–62.91% of them were women, mean age (SD) was 55.65 (7.55)–55.72 (7.54) years. 38,309 participants answered maternal smoking during pregnancy-related questions, 63.05% of them were women, mean age (SD) was 55.47 (7.55) years. Other details were shown in Table 1.

**Table 1:** Descriptive characteristics of study participants.

Outcome Variable	Instrumental variable	Number	Sex (Female)	Age ± SD
	Felt hated by family member	40,112	24,880(62.03%)	55.72 ± 7.54
	Physically abused by family	40,464	25,454(62.91%)	55.71 ± 7.54
Age at onset of depression	Felt loved	35,633	22,321(62.64%)	55.66 ± 7.56
	Sexually molested	41,595	25,754(61.92%)	55.65 ± 7.55
	Mother smoking	38,309	24,152(63.05%)	55.47 ± 7.55
	Depression PRS	43,664	27,358(62.66%)	55.63 ± 7.55

### Associations between age at onset of the depression and early life factors × depression PRS

In the regression analysis for all participants, we found that age at onset of the depression was significantly associated with the 4 childhood traumatic events, such as, felt hated by family member as a child (*β* = −7.385, *P* = 2.00 × 10^−16^), and felt loved as a child (*β* = 5.534, *P* = 2.00 × 10^−16^). We also found age at onset of the depression was significantly associated with the interaction between felt loved as a child and depression PRS (*β* = 0.708, *P* = 5.03 × 10^−3^). The details were shown in Table 2.

**Table 2:** Association between Age at onset of depression and early life factors during childhood, Depression PRS.

Outcome variable	Environment factor	Instrumental variable											
		Environment factor				Depression PRS				Environment factor * Depression PRS			
		Beta	SE	*T*	*P*-value	Beta	SE	*T*	*P*-value	Beta	SE	*T*	*P*-value
Age at onset of depression for all participants	Felt hated by family member	−7.385	0.379	−19.506	2.00 × 10^−16^	−0.287	0.370	−0.776	4.38 × 10^−1^	0.033	0.347	0.094	9.25 × 10^−1^
	Physically abused by family	−4.538	0.552	−8.217	2.00 × 10^−16^	0.251	0.533	0.470	6.38 × 10^−1^	−0.550	0.518	−1.062	2.88 × 10^−1^
	Felt loved	5.534	0.271	20.446	2.00 × 10^−16^	−1.601	0.485	−3.304	9.55 × 10^−4^	0.708	0.252	2.806	5.03 × 10^−3^
	Sexually molested	−5.028	0.683	−7.367	1.77 × 10^−13^	0.102	0.570	0.179	8.58 × 10^−1^	−0.383	0.556	−0.689	4.91 × 10^−1^
	Mother smoking	−0.087	0.159	−0.547	5.84 × 10^−1^	−0.326	0.224	−1.458	1.45 × 10^−1^	−0.001	0.160	−0.008	9.94 × 10^−1^
Age at onset of depression for females	Felt hated by family member	−7.805	0.445	−17.523	2.00 × 10^−16^	−0.438	0.442	−0.991	3.22 × 10^−1^	0.101	0.411	0.245	8.06 × 10^−1^
	Physically abused by family	−5.087	0.692	−7.348	2.07 × 10^−13^	−0.241	0.686	−0.351	7.26 × 10^−1^	−0.123	0.667	−0.184	8.54 × 10^−1^
	Felt loved	6.352	0.341	18.610	2.00 × 10^−16^	−0.742	0.603	−1.230	2.19 × 10^−1^	0.232	0.314	0.740	4.60 × 10^−1^
	Sexually molested	−4.975	0.732	−6.801	1.06 × 10^−11^	−0.001	0.611	−0.001	9.99 × 10^−1^	−0.340	0.591	−0.576	5.64 × 10^−1^
	Mother smoking	−0.224	0.199	−1.123	2.61 × 10^−1^	−0.317	0.279	−1.133	2.57 × 10^−1^	−0.039	0.201	−0.194	8.47 × 10^−1^
Age at onset of depression for males	Felt hated by family member	−6.270	0.713	−8.795	2.00 × 10^−16^	0.023	0.674	0.035	9.72 × 10^−1^	−0.131	0.641	−0.204	8.38 × 10^−1^
	Physically abused by family	−3.553	0.914	−3.888	1.01 × 10^−4^	1.033	0.848	1.219	2.23 × 10^−1^	−1.207	0.821	−1.470	1.42 × 10^−1^
	Felt loved	4.181	0.444	9.424	2.00 × 10^−16^	−2.860	0.812	−3.525	4.26 × 10^−4^	1.421	0.422	3.366	7.64 × 10^−4^
	Sexually molested	−5.574	1.911	−2.916	3.55 × 10^−3^	0.012	1.667	0.007	9.94 × 10^−1^	−0.188	1.655	−0.114	9.10 × 10^−1^
	Mother smoking	0.212	0.263	0.808	4.19 × 10^−1^	−0.316	0.373	−0.849	3.96 × 10^−1^	0.046	0.265	0.175	8.61 × 10^−1^

In the regression analysis for all females, we found that age at onset of the depression was significantly associated with the 4 childhood traumatic events, such as felt hated by family member as a child (*β* = −7.805, *P* = 2.00 × 10^−16^), felt loved as a child (*β* = 6.352, *P* = 2.00 × 10^−16^), but there is no significant association for age at onset of the depression and interaction between early life factors and depression PRS. The details were shown in Table 2.

In the regression analysis for all males, we found that age at onset of the depression was significantly associated with the 4 childhood traumatic events, such as felt hated by family member as a child (*β* = −6.270, *P* = 2.00 × 10^−16^), felt loved as a child (*β* = 4.181, *P* = 2.00 × 10^−16^). Besides, we found age at onset of the depression was significantly associated with the interaction between felt loved as a child and depression PRS (*β* = 1.421, *P* = 7.64 × 10^−4^). The details were shown in Table 2.

### GWEIS analysis results

**Fig. 1:**
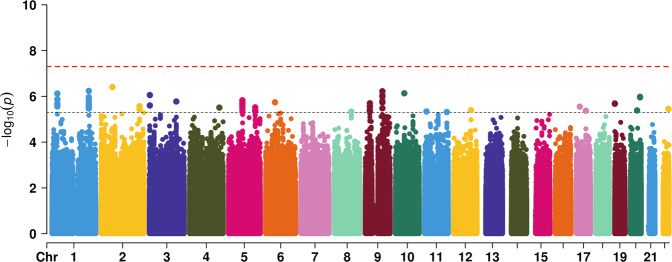
Manhattan plot for GWEIS analysis in all participants. Genomic coordinates are displayed along the *X*-axis, with the negative logarithm of the association *P* value for each SNP displayed on the *Y*-axis, meaning that each dot on the Manhattan plot signifies a SNP. The red line indicates the *P*-value threshold for genome-wide significance (*P* = 5 × 10^−8^) while the blue line indicates *P*-value threshold for suggestive significance (*P* = 5 × 10^−6^).

For significant interaction results in regression analysis, we further performed GWEIS analysis. In the regression analysis containing all participants, the interaction between felt loved as a child and depression PRS for age at onset of depression was significant. GWEIS identified some suggestive significant genes. The visualization of the results was provided in Fig. 1 and Table 3.

**Table 3:** The summary of genetic variants interacting with felt loved as a child for age at onset of depression in all participants.

SNP	CHR	Gene	Model	OR	*P*
rs4744313	9	*PTPDC1*	ADD × felt loved	4.99	5.99 × 10^–7^
rs10821292	9	*PTPDC1*	ADD × felt loved	4.91	8.97 × 10^–7^
rs10114320	9	*PTPDC1*	ADD × felt loved	4.89	1.03 × 10^–6^
rs10993052	9	*PTPDC1*	ADD × felt loved	4.80	1.61 × 10^–6^
rs6902510	6	*LINC01564*	ADD × felt loved	−4.77	1.82 × 10^–6^
rs7343	5	*CMYA5*	ADD × felt loved	4.75	2.03 × 10^–6^
rs2079157	19	*ARHGAP45*	ADD × felt loved	4.75	2.06 × 10^–6^
rs72975514	19	*ARHGAP45*	ADD × felt loved	4.74	2.11 × 10^–6^
rs3749682	5	*CMYA5*	ADD × felt loved	4.73	2.21 × 10^–6^
rs207879	2	*XRCC5*	ADD × felt loved	−4.69	2.69 × 10^–6^
rs71620487	4	*ANXA10*	ADD × felt loved	−4.66	3.10 × 10^–6^
rs4282632	9	*PTPDC1*	ADD × felt loved	4.65	3.33 × 10^–6^
rs35784919	5	*ANXA10*	ADD × felt loved	4.64	3.43 × 10^–6^
rs828704	2	*XRCC5*	ADD × felt loved	−4.63	3.64 × 10^–6^
rs7214413	17	*ANKFN1*	ADD × felt loved	−4.60	4.25 × 10^–6^
rs3134506	8	*C8orf37-AS1*	ADD × felt loved	4.58	4.66 × 10^–6^
rs535603	11	*KIRREL3*	ADD × felt loved	−4.57	4.84 × 10^–6^
rs557008	11	*KIRREL3*	ADD × felt loved	−4.57	4.84 × 10^–6^

Abbreviation: *SNP* single nucleotide polymorphism; *CHR* chromosome.

**Fig. 2:**
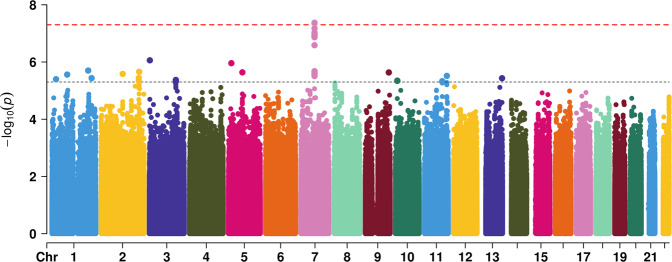
Manhattan plot for GWEIS analysis in males. Genomic coordinates are displayed along the *X*-axis, with the negative logarithm of the association *P* value for each SNP displayed on the *Y*-axis, meaning that each dot on the Manhattan plot signifies a SNP. The red line indicates the *P*-value threshold for genome-wide significance (*P* = 5 × 10^−8^) while the blue line indicates *P*-value threshold for suggestive significance (*P* = 5 × 10^−6^).

Besides, the interaction between felt loved as a child and depression PRS for age at onset of the depression was also significant in males. GWEIS detected a candidate loci interacting with felt loved as a child at *GSAP* (rs2068031, *P* = 4.24 × 10^–8^) in males. Additionally, we detected several genes with suggestive associations, such as *PTPDC1* (rs4744313, *P* = 5.99 × 10^–7^), *KIRREL3* (rs535603, *P* = 4.84 × 10^–6^) and *CMYA5* (rs7343, *P* = 2.03 × 10^–6^). The visualization of the results was provided in Fig. 2 and Table 4.

**Table 4:** The summary of genetic variants interacting with felt loved as a child for age at onset of depression in males.

SNP	CHR	Gene	Model	OR	*P*-value
rs2068031	7	*GSAP*	ADD × felt loved	−5.48	4.24 × 10^–8^
rs4744313	9	*PTPDC1*	ADD × felt loved	4.99	5.99 × 10^–7^
rs10821292	9	*PTPDC1*	ADD × felt loved	4.91	8.97 × 10^–7^
rs10114320	9	*PTPDC1*	ADD × felt loved	4.89	1.03 × 10^–6^
rs10993052	9	*PTPDC1*	ADD × felt loved	4.80	1.61 × 10^–6^
rs6902510	6	*LINC01564*	ADD × felt loved	−4.77	1.82 × 10^–6^
rs7343	5	*CMYA5*	ADD × felt loved	4.75	2.03 × 10^–6^
rs2079157	19	*ARHGAP45*	ADD × felt loved	4.75	2.06 × 10^–6^
rs72975514	19	*ARHGAP45*	ADD × felt loved	4.74	2.11 × 10^–6^
rs3749682	5	*CMYA5*	ADD × felt loved	4.73	2.21 × 10^–6^
rs207879	2	*XRCC5*	ADD × felt loved	−4.69	2.69 × 10^–6^
rs397387	5	*LINC01933*	ADD × felt loved	−4.66	3.03 × 10^–6^
rs71620487	4	*ANXA10*	ADD × felt loved	−4.66	3.10 × 10^–6^
rs4282632	9	*PTPDC1*	ADD × felt loved	4.65	3.33 × 10^–6^
rs35784919	5	*CMYA5*	ADD × felt loved	4.64	3.43 × 10^–6^
rs828704	2	*XRCC5*	ADD × felt loved	−4.63	3.64 × 10^–6^
rs167193	5	*LINC01933*	ADD × felt loved	−4.61	4.04 × 10^–6^
rs7214413	17	*ANKFN1*	ADD × felt loved	−4.60	4.25 × 10^–6^
rs61632389	5	*LINC01933*	ADD × felt loved	−4.59	4.36 × 10^–6^
rs159229	5	*LINC01933*	ADD × felt loved	−4.59	4.44 × 10^–6^
rs3134506	8	*C8orf37-AS1*	ADD × felt loved	4.58	4.66 × 10^–6^
rs535603	11	*KIRREL3*	ADD × felt loved	−4.57	4.84 × 10^–6^
rs557008	11	*KIRREL3*	ADD × felt loved	−4.57	4.84 × 10^–6^

Abbreviation: *SNP* single nucleotide polymorphism; *CHR* chromosome.

## Discussion

Environmental exposures early in development have a role in susceptibility to disease in later life [24]. Early life events can be associated with permanent changes in physiology and/or structure [25]. Many of these changes are associated with permanent alterations in gene expression regulated by epigenetic factors such as DNA methylation and histone methylation/acetylation [25]. In mammals, there are two major reprogramming periods, where the epigenome is erased and then re-established: gametogenesis and embryo-foetal development [26]. This early-life period is therefore characterized by a remarkable epigenetic plasticity [26]. Subsequently, the individual’s environment could influence its epigenome, leading to a vicious circle involved in the development of pathologies [26]. Childhood is recognized as a window of vulnerability and opportunity [27]. The age at onset of depression is an important cause that is related to the course and symptomatology of depressive episode determined by a number of genetic and environmental factors [17, 28, 29]. In this study, we combined the genetic factors and early life factors for age at onset of depression through conducting GWEIS to clarify the potential effects of SNP × early life factors interaction.

In the observational study, we found associations between age at onset of depression and felt loved as a child × depression PRS. The parent-adolescent relationship is a primary source of love [30]. Emotional abuse and neglect mean the failure to provide a supportive environment by, for example, verbally threatening the child, or the failure to provide for all aspects of the child’s well-being [31]. It has been reported by previous studies that the role of neglect and emotional abuse is significantly associated with depression [32]. Neglect and emotional abuse may impede a proper development of emotional processes in the child with an increased risk to develop depressive states in adulthood [33]. The same as our findings, familial emotional support, like felt loved as a child is inversely related to the age of onset of depression.

Besides, to the best of our knowledge, there are limited researches to explore the genetic mechanism affecting the link between felt loved as a child and the age of onset of depression. The treatment response of mood disorder patients with a history of child abuse and neglect has a more severe clinical course in terms of symptom severity and age of onset, and responds more poorly to pharmacotherapy and/or psychotherapy [34]. Thus, we carried out the GWEIS of the age at onset of depression as key point of this research and identified multiple loci and a significant gene for the regulation of genetic response to traumatic events—felt loved as a child during childhood, providing novel clues to help disentangle its underlying etiology. GWEIS identified a candidate loci interacting with felt loved as a child for age at onset of depression located on gene, *GSAP*. SNP located in the *GSAP* promoter region comprises an allele associated with high *GSAP* expression, which correlates with increased Alzheimer’s disease (AD) risk [35]. Previous study found late-onset treatment-resistant depression was associated with an elevated risk of dementia and AD [36]. A growing number of evidence have revealed that depression, particularly late-life depression, could be a prodrome of dementia or AD [36, 37]. Biochemical, pathogenic, and human genetic data confirm *GSAP* is a selective γ-secretase modulatory protein [38]. The inhibition of γ-secretase activity could rescue memory impairment by attenuating the generation of Aβ [39].

We also found some genes with suggestive significance. For example, *KIRREL3* is important for the development of nervous system, in particular for the process of neuronal migration, axonal fasciculation, and synapse formation [40]. Alterations of *KIRREL3* have been linked to neurodevelopmental disorders such as intellectual disability, autism spectrum disorder, and bipolar disorder [41]. In the hippocampus, *KIRREL3* is highly expressed in dentate granule neurons and scattered GABAergic neurons of the hilus and CA3 [42]. In animal models, adult male rats exposed to chronic social isolation show depressive- and anxiety-like behaviors and reduce the numbers of parvalbumin-positive interneurons in the dorsal hippocampus[43]. However, it was found that tianeptine could offer protection from chronic social isolation via modulation of the dorsal hippocampal GABAergic system [43].

Further, *CMYA5* was also detected. Previous study found that *CMYA5* confer genetic risk for both schizophrenia and MDD [44]. They detected rs7343 was associated with MDD significantly [44]. Rs7343, a locus was also observed in our study, located in the 3′ UTR region of *CMYA5* where sequence variations might alter the microRNAs binding sites [44]. Besides, they revealed hsa-mir-659 is possible to form a miRNA/SNP target duplex with the A allele of CYMA5 [44], and miR-659 is confirmed to be expressed in human brain [45]. CMYA5 and its gene product, myospryn, in the brain and neuronal cells with its binding partner, desmin, an intermediate filament protein may provide structural support and efficient rearrangement of the cytoskeleton network during early neuritogenesis [46].

In the observational and GWEIS analyses, we also found gender differences for the age at onset of depression and felt loved as a child × depression associations. A previous twin study found although women had a higher frequency of depressive symptoms and depression diagnoses, depressive symptoms and diagnosis of depression increased slightly more among men over time [47]. Besides, male adolescents reported significantly higher levels of emotional and physical neglect, and external-oriented thinking style than female adolescents [48]. In animal models, it was found a decrease in serotonin in amygdala induced by maternal separation in male rats [49]. Serotonin is a neurotransmitter associated to physiological and behavioral functions, such as motor activity, hormone secretion, mood, and cognition. The decrease in serotoninergic activity in the entire amygdala is associated with an increase in anxiety-like behavior [49]. Therefore, this study identified significant gender differences.

To the best of our knowledge, this is a novel study based on a large cohort study with a long follow-up as well as representative samples. Besides, we calculated depression PRS, explored the genetic associations between depression and traits, and assessed the influence of susceptible loci on disease risks. The depression PRS acted as genetic background for age at onset of the depression interacting with early life factors to access the joint effect of genetic and environmental risk factors for age at onset of the depression in individuals. These may give insights into the mechanism of depression. However, there are still some limitations. First, owing to all samples in this study is from European ancestry, the findings should be inferred to other races with caution. Second, our study results should be interpreted with caution and further studies are needed to confirm the findings and reveal the potential roles of identified loci in the development of age at onset of the depression.

In summary, through observational and GWEIS analyses, this study indicated emotional care in childhood may affect the age at onset of depression, especially in males, and further identified *GSAP* as a candidate gene. Identifying the significant gene × environment interactions underlying the age at onset of depression could help to reduce the incidence and mortality of depression and other related psychiatric disorders.

## Data Availability

The UKB data are available through the UK Biobank Access Management System (https://www.ukbiobank.ac.uk/). We will return the derived data fields following UKB policy; in due course, they will be available through the UK Biobank Access Management System.
